# Supplementary material for: Knowledge, attitude, and practice towards COVID-19 and associated factors among students in Ethiopia: Systematic review and meta-analysis
Source: PLoS One. 2024 Dec 9;19(12):e0314451. doi: 10.1371/journal.pone.0314451 (PMC11627395; doi:10.1371/journal.pone.0314451)
Supplement: S2 Table — (PDF) [file pone.0314451.s002.pdf]

S2 Table: search strategies for different data bases.

Data base	Search	Search term
PubMed	#1	((knowledge) OR (awareness)) OR (Epistemology) AND ((ffrft[Filter]) AND (humans[Filter]) AND (english[Filter]))
	#2	((Attitude) OR (Opinions)) OR (Sentiment) AND ((ffrft[Filter]) AND (humans[Filter]) AND (english[Filter]))
	#3	((((((Practice*) OR ("Prevent* practice*")) OR ("Prevent* behavior*")) OR ("Practice* prevent* measure*")) OR ("self-protective practice*")) OR ("Adherence prevent* measure*")) OR ("Adherence prevent* behavior*") AND ((ffrft[Filter]) AND (humans[Filter]) AND (english[Filter]))
	#4	(((((((((((((COVID-19) OR ("2019 Novel Coronavirus Disease")) OR ("2019 Novel Coronavirus Infection")) OR ("2019-nCoV Disease")) OR ("2019-nCoV Infection")) OR ("COVID-19 Pandemic*")) OR ("COVID-19 Virus Disease")) OR ("COVID-19 Virus Infection")) OR ("SARS-CoV-2")) OR ("Coronavirus Disease 2019")) OR ("Coronavirus Disease-19")) OR ("SARS Coronavirus 2 Infection")) OR ("SARS-CoV-2 Infection")) OR ("Severe Acute Respiratory Syndrome Coronavirus 2 Infection")) OR ("Coronavirus infections")
	#5	("associated factor*"[Mesh Terms]) OR (determinant*[Mesh Terms]) OR (factor*[Mesh Terms]) OR (predictor*[Mesh Terms]) OR ("risk factor*"[Mesh Terms])
	#6	(((((((((((Student*) OR ("School Enrollment*")) OR (learner*)) OR ("School boy")) OR ("School girl")) OR (undergraduate)) OR (graduate)) OR (postgraduate)) OR (freshman)) OR ("Medical Student*")) OR ("Non-medical student*")) OR ("Preparatory student*")) OR ("Secondary student*") AND ((ffrft[Filter]) AND (humans[Filter]) AND (english[Filter]))
	#7	((Ethiopia) OR ("Federal Democratic Republic of Ethiopia")) OR (Abyssinia) AND ((ffrft[Filter]) AND (humans[Filter]) AND (english[Filter]))
	#8	#1OR#2OR#3
	#9	#8OR#5AND#4
	#10	#9AND#6AND#7
Google Scholar, African Journals Online, Research for life, and Science Direct	#1	Knowledge OR Attitude OR Practice OR associated factor OR determinant OR factor OR risk factor AND Coronavirus Disease-19 AND Student AND Ethiopia
