# Supplementary material for: Knowledge, attitude, and practice towards COVID-19 and associated factors among students in Ethiopia: Systematic review and meta-analysis
Source: PLoS One. 2024 Dec 9;19(12):e0314451. doi: 10.1371/journal.pone.0314451 (PMC11627395; doi:10.1371/journal.pone.0314451)
Supplement: S3 Table — (PDF) [file pone.0314451.s003.pdf]

S3 Table: All studies identified and Quality appraisal

Joanna Bridges Institute (JBI) grading approach for observational studies										
	**Authors, year**	**Q1**	**Q2**	**Q3**	**Q4**	**Q5**	**Q6**	**Q7**	**Q8**	**Quality level**
1	Yesuf M and Abdu M (2022)	Y	Y	N	Y	Y	N	Y	Y	Good
2	Tadesse AW et al (2020) https://ethjhealths.org/preventive-practice-and-associated-factors-towards-covid-19-among-college-students-amhara-region	Y	Y	N	Y	Y	Y	Y	Y	Good
3	Tazebew B et al (2023) https://go.exlibris.link/p1g73Nkj	N	Y	N	Y	Y	N	Y	Y	Good
4	Getawa S et al (2022)	Y	Y	N	Y	Y	N	Y	Y	Good
5	Berihun G et al (2021)	Y	Y	N	Y	Y	N	Y	Y	Good
6	Mekonnen A (2021) http://etd.aau.edu.et/handle/123456789/29006	N	Y	N	Y	Y	N	Y	Y	Good
7	Aynalem YA et al (2021)	N	Y	N	Y	Y	Y	Y	Y	Good
8	Angelo AT et al (2020)	N	Y	N	Y	Y	N	Y	Y	Good
9	Walle TA and Temachu YZ (2023)	N	Y	N	Y	Y	Y	Y	Y	Good
10	Feleke A et al (2022)	N	Y	N	Y	Y	N	Y	Y	Good
11	Gutata D et al (2023) https://scienceopen.com/hosted-document?doi=10.14293/S2199-1006.1.SOR-.PPXLOI0.v1	N	Y	N	Y	Y	N	Y	Y	Good
12	Handebo S et al (2021)	N	Y	N	Y	Y	N	Y	Y	Good
13	Asefa L et al (2021) https://www.researchsquare.com/article/rs-628626/v1	N	N	N	Y	Y	Y	Y	Y	Good
14	Tsegaw M et al (2022)	N	Y	N	Y	Y	N	Y	Y	Good
15	Wogayehu B et al (2020) https://doi.org/10.21203/rs.3.rs-24777/v1	Y	Y	N	Y	Y	N	Y	Y	Good
16	Feleke A et al (2022)	N	Y	N	Y	Y	N	Y	Y	Good
17	Tadese M et al (2022)	N	N	N	Y	Y	N	Y	Y	Good
18	Tadese M et al (2021)	N	N	N	Y	Y	N	Y	Y	Good
19	Kebede BF et al (2022) https://doi.org/10.1371/journal.pone.0279081	N	N	Y	Y	N	N	Y	Y	Poor (Excluded due to quality)
20	Birhanu Z et al (2022) https://doi.org/10.3389/fpubh.2022.1082563	Excluded due to outcome difference								
21	Shitu K et al (2022) https://doi.org/10.1371/journal.pone.0263568	Excluded due to outcome difference								

Note: Y=Yes, N= No
