# Supplementary material for: Knowledge, attitude, and practice towards COVID-19 and associated factors among students in Ethiopia: Systematic review and meta-analysis
Source: PLoS One. 2024 Dec 9;19(12):e0314451. doi: 10.1371/journal.pone.0314451 (PMC11627395; doi:10.1371/journal.pone.0314451)
Supplement: Attachment — Submitted filename: Response to Reviewers.pdf [file pone.0314451.s004.pdf]

<u>RESPONSE TO REVIEWERS</u>

Submission ID: PONE-D-24-12869

Title: Knowledge, attitude, and practice towards COVID-19 and associated factors among students in Ethiopia: Systematic review and meta-analysis

Journal Name: PLOS ONE

Academic Editor: Festo Casmir Shayo, M.D, M.Med., PhD

Date: 10/10/2024

Dear editors and reviewers

Thank you for providing valuable comments, suggestions, and coordination to improve the quality of our manuscript. As per your request, we have made appropriate revisions, which are highlighted in green in the 'Revised Manuscript with Track Changes' document. The original document titled 'manuscript' has been submitted separately. We have also provided a point-by-point response to each reviewer's and editor's comments, with page numbers referring to the revised manuscript file. We are looking forward to the outcome of your assessment. Thank you for your valuable time and consideration.

Academic editor comments

1. ***In Tables 2 to 6: Please remove all vertical lines and retain only three horizontal lines: one under the title, one above the column heading, and one between the column headings and the body of the table***

   Authors' response: Thank you for your comment, correction was made from Table 2 to 6.

2. ***Please revise your references to make sure that they follow the PLOS ONE referencing style***

   Authors' response: Thank you for your comment. The comment is correct, and the necessary correction has been made.

3. ***It is encouraged to have your manuscript undergo a professional English language edition to suit publication standards***

   Authors' response: Thank you for your comment. The comment is correct, and the necessary correction has been made.

   <u>Reviewers' comments:</u>

Reviewer's Responses to Questions

Comments to the Author

1. Is the manuscript technically sound, and do the data support the conclusions?

The manuscript must describe a technically sound piece of scientific research with data that supports the conclusions. Experiments must have been conducted rigorously, with appropriate controls, replication, and sample sizes. The conclusions must be drawn appropriately based on the data presented.

Reviewer #1: Partly

Authors' response: Thank you for your comment, we corrected have the conclusion part.

2. Has the statistical analysis been performed appropriately and rigorously?

Reviewer #1: Yes

Authors' response: Thank you for your comment.

3. Have the authors made all data underlying the findings in their manuscript fully available?

Reviewer #1: Yes

Authors' response: Thank you for your comment.

4. Is the manuscript presented in an intelligible fashion and written in standard English?

Reviewer #1: No

Authors' response: Thank you for your comment. The comment is correct, and the necessary correction has been made.

5. Review Comments to the Author

1. Reviewer #1: L1: The title can be improved by adding "associated factors" instead of "predictors" given the cross-sectional nature of included studies instead of cohort studies.

Authors' response: Thanks for your comment. The reviewer was right, and the change was made on page 1.

Abstract

2. ***L23-24: Add "s" to "pooled proportion"; "positive" before "attitude" and "prevention" before "practice"***

Authors' response: Thanks for your comment, the reviewer was right, and the change was made on page 2.

3. ***L32: Add "s" to "pooled proportion"; "positive" before "attitude" and "prevention" before "practice"***

Authors' response: Thanks for your comment, the reviewer is right, and the change was made on page 2.

Introduction

4. ***L71: Add a preposition "in" after "results"***

Authors' response: Thanks for your comment, the reviewer is right, and the change was made on page 4.

5. ***L74: Add "s" to "pooled proportion"***

Authors' response: Thanks for your comment, the reviewer is right, and the change was made on page 4.

Methods

6. ***L83: Add "an" before amendment; "made" instead of "done"; and "an" before additional outcome.***

Authors' response: Thanks for your comment, the reviewer is right, and the change was made on page 5.

7. ***L93-94: Rewrite the sentence "In addition, articles included were cross-sectional study design written only in English until December 10, 2023." to improve grammar.***

Authors' response: Thanks for your comment, the reviewer is right, and the change was made on page 5.

Discussion

8. ***L236: The term "in the world" after the word "pandemic" is redundant. Please remove it.***

Authors' response: Thanks for your comment; correction was made on page 17.

9. ***L237-238: Change the order of words in "a lot of people have died and suffered from it": suffer to come before death.***

Authors' response: Thanks, the reviewer is right and the correction was made on page 17.

10. ***L238-239: "The World Health Organization (WHO) does not … or the invention of the drug" is not clear. Please rewrite to improve clarity.***

Authors' response: Thanks for your comment; correction was made on page 17.

11. ***L239-240: The sentence "The best management for the disease is relieving the symptoms, not the virus" needs to be rewritten.***

Authors' response: Thanks for your comment; correction was made on page 17.

12. ***L241-243: The sentence "Regular studies are needed to perform any intervention, advocacy, and health education and communication program, even to change knowledge,***

attitude, and practice regarding COVID-19 among students in Ethiopia" needs to be rewritten.

Authors' response: Thanks for your comment; correction was made on page 17.

13. L243: The sentence "Due to scarcity among students . . ." is not clear. Please rewrite.

Authors' response: Thanks for your comment; correction was made on page 17.

Strengths and limitations

14. The included studies assessed adequate knowledge, positive attitude, and prevention practice by different sets of questions formulated by respective authors and not standardized. This variability in the assessment criteria affects pooling. I suggest this to be considered as a limitation.

Authors' response: Thanks for your comment, the reviewer is right. Correction was made on page 21.

15. L318: Use the word "generalizability" instead of "generalization".

Authors' response: Thanks for your comment; Correction was made on page 21.

Conclusions

16. L327-328: Rewrite the sentence "The pooled proportion of attitudes and practices eradicated in 2021 from 2020 year indicates the need for more intervention, advocacy, and policy change"

Authors' response: Thanks for your comment; Correction was made on page 21.

17. L329: Add "positive" before "attitudes".

Authors' response: Thanks for your comment; Correction was made on page 21.

18. L330: "The result also pointed out the need to give attention for male" should be rewritten as "attention to" "females" instead of "males" as per your results.

Authors' response: Thanks for your comment; Correction was made on page 21.

References

19. Many of the references need to be rewritten.

Authors' response: Thanks for your comment, the reviewer is right, and the change was made.

20. Fig 3 and 6 headings: add "positive" before "attitude".

Authors' response: Thanks for your comment, the reviewer is right and the correction was made on Fig 3 and Fig 6.
